# Small studies, big decisions: the role of pilot/feasibility studies in incremental science and premature scale-up of behavioral interventions

**DOI:** 10.1186/s40814-021-00909-w

**Published:** 2021-09-10

**Authors:** Michael W. Beets, Lauren von Klinggraeff, R. Glenn Weaver, Bridget Armstrong, Sarah Burkart

**Affiliations:** Arnold School of Public Health, University of South Carolina, Columbia, USA

**Keywords:** Scaling, Translation, Intervention, Pilot, Feasibility, Early-stage

## Abstract

**Background:**

Careful consideration and planning are required to establish “sufficient” evidence to ensure an investment in a larger, more well-powered behavioral intervention trial is worthwhile. In the behavioral sciences, this process typically occurs where smaller-scale studies inform larger-scale trials. Believing that one can do the same things and expect the same outcomes in a larger-scale trial that were done in a smaller-scale preliminary study (i.e., pilot/feasibility) is wishful thinking, yet common practice. Starting small makes sense, but small studies come with big decisions that can influence the usefulness of the evidence designed to inform decisions about moving forward with a larger-scale trial. The purpose of this commentary is to discuss what may constitute sufficient evidence for moving forward to a definitive trial. The discussion focuses on challenges often encountered when conducting pilot/feasibility studies, referred to as common (mis)steps, that can lead to inflated estimates of both feasibility and efficacy, and how the intentional design and execution of one or more, often small, pilot/feasibility studies can play a central role in developing an intervention that scales beyond a highly localized context.

**Main body:**

Establishing sufficient evidence to support larger-scale, definitive trials, from smaller studies, is complicated. For any given behavioral intervention, the type and amount of evidence necessary to be deemed sufficient is inherently variable and can range anywhere from qualitative interviews of individuals representative of the target population to a small-scale randomized trial that mimics the anticipated larger-scale trial. Major challenges and common (mis)steps in the execution of pilot/feasibility studies discussed are those focused on selecting the right sample size, issues with scaling, adaptations and their influence on the preliminary feasibility and efficacy estimates observed, as well as the growing pains of progressing from small to large samples. Finally, funding and resource constraints for conducting informative pilot/feasibility study(ies) are discussed.

**Conclusion:**

Sufficient evidence to scale will always remain in the eye of the beholder. An understanding of how to design informative small pilot/feasibility studies can assist in speeding up incremental science (where everything needs to be piloted) while slowing down premature scale-up (where any evidence is sufficient for scaling).

## Background

Behavioral scientists place a strong emphasis on the importance of scaling interventions [1–4]. Scaling is defined as increasing the size, reach, or impact of health interventions and occurs anywhere along the translational pipeline, from the initial development, up through the national/global dissemination of an intervention [5–7]. The term scaling is often used to describe the end of the translational pipeline where interventions found to be efficacious in a local setting are translated for broader population-level impact. Although less widely used, scaling can describe the processes occurring during intervention development where initial early-stage pilot/feasibility studies are conducted to inform a subsequent larger trial of a behavioral intervention [8].

Almost every large-scale, well-powered trial is informed by one or more pilot/feasibility studies [9]. Preliminary, early-stage studies are designed to generate sufficient evidence to make informed decisions by the investigative team and grant funding agencies whether an intervention is promising and the findings (both feasibility and initial evidence of efficacy) from the preliminary study can be replicated in a larger, more well-powered trial (i.e., scaled). Replication in this sense refers broadly to the ability to recruit the target population, deliver the intervention with some degree of fidelity, measure primary/secondary outcomes, and establish initial evidence of efficacy in changing outcomes that are demonstrated within the pilot/feasibility study, in the larger-scale trial. Thus, the decisions to scale often require evidence to be gathered to allow for an evaluation of the scientific merit of both impact and implementation of an intervention, with evidence gathered and presented within publications/grant applications about *trial feasibility*, recruitment of the target population and assessment of outcomes; *intervention implementation and feasibility*, participant enjoyment/acceptability, attendance/dosage, and missing or needed intervention components; and *preliminary efficacy*, whether an initial clinically significant “signal” of promise is detected via changes in either primary and/or secondary outcomes [10–12].

The ability to establish “sufficient” evidence to support a larger-scale, more well-powered, definitive trial, from smaller studies, however, is complicated. For any given intervention, the type and amount of evidence necessary to be deemed “sufficient” is inherently variable and can range anywhere from qualitative interviews to a small-scale randomized trial that mimics the anticipated larger, scaled trial. In some instances, feasibility related information may be sufficient (e.g., we can recruit the target population, the target population “likes” the intervention components), whereas in others, evidence indicating changes in primary or secondary outcomes may be necessary. It is not uncommon for a preliminary study to exclusively focus on markers of trial and implementation feasibility, and then be followed by an additional preliminary study evaluating whether a change in a clinically significant signal occurs. This variability makes the evaluation of the products (e.g., interviews, recruitment rates, satisfaction, implementation, changes in outcomes/preliminary clinically significant signal of promise) from one or more pilot/feasibility studies challenging, with the judgment of “sufficiency” ultimately left to be determined by those that produce the evidence (i.e., intervention team) as well as those that evaluate the evidence (e.g., grant panel members).

A challenge when determining markers of “sufficiency” is the need to ensure that innovation is not inadvertently slowed down, yet appropriately requires a standard of evidence to be available that minimizes scaling-up too quickly. The balance between incremental science and premature scale-up is important given the resource constraints for pilot/feasibility studies and the need to invest substantial sums of monies in interventions least likely to fail when scaled. Existing frameworks describe a flexible, iterative series of studies designed to inform scaling decisions of behavioral interventions [13, 14]. Embedded within these are cautionary notes of scaling too quickly prior to establishing “sufficient” evidence that an intervention has impact and can be implemented and that the intervention can reasonably be transferred (i.e., scaled) to the next subsequent, often larger trial. It is potentially more efficient and cost effective to design and test aspects of feasibility and preliminary efficacy in one or more preliminary studies in order to identify early warning signs forecasting problems with scaling, rather than encountering challenges in the larger-scale trial that could have been readily addressed during early-stage studies.

In this commentary, we discuss the big decisions interventionists are faced with in the design and interpretation of feasibility and preliminary efficacy outcomes to scale an intervention from a smaller-scale pilot/feasibility study to a larger, more well-powered trial. We review common challenges and (mis)steps in pilot/feasibility studies that can lead to inflated estimates of initial promise of an intervention and the premature scale-up of behavioral interventions. The commentary reviews key characteristics of pilot/feasibility studies that, when judged separately and collectively, can lead to informed decisions about whether a behavioral intervention is ready to be scaled (for the interventionists) or the investment in the scaling is justified (for grant review panels). The intention of this commentary is to answer the following overarching questions faced by intervention developers and reviewers when designing, conducting, and reviewing pilot/feasibility studies:

When designing a pilot/feasibility study, what should one do to ensure sufficient evidence to scale is collected?

When reviewing pilot/feasibility products, what should be considered sufficient evidence to warrant scaling?

## Challenge — what is an appropriate sample size of a pilot/feasibility study?

Common sense and current practice tell us early-stage, preliminary pilot/feasibility studies are smaller than the larger, more well-powered trial they inform. In the behavioral sciences, recruiting participants and delivering an intervention is resource intensive and this alone is a likely driver of smaller samples during early-stage testing. Prior reviews indicate the median sample size of pilot studies is ~ 30 participants per arm [15]. Others define pilot studies as those with 100 or fewer total participants [16]. In a recent meta-epidemiological study of pilot studies that scaled to a well-powered, larger-scale trial, the median sample size of the preliminary studies was 61 with over two thirds of the pilot studies containing 100 or fewer total participants [8].

Intuitively, starting small makes sense [17]. Smallness allows for greater agility, fewer resources, and lower stakes for failure than commencing with a large-scale trial of an untested intervention. Smallness accelerates the understanding of what does and does not work in behavioral interventions allowing for modifications to be made and where necessary re-piloting prior to embarking with the larger-scale trial. Hence, there are numerous benefits and justifiable reasons for starting small. Given this, what should the sample size be? In the context of pilot/feasibility studies, sample size does not refer to power, especially at the early stages of testing. If a pilot study is sufficiently powered, then it ceases to be a pilot study. The appropriate sample size, therefore, must be justified based on an understanding of the form and function of how an intervention may operate under the conditions at-scale. This implies investigators have a working knowledge of the homo/heterogeneity in the target population, the setting(s) and delivery of the intervention, and how variations in one or more of these can lead to increased/decreased changes in the outcomes of interest. With this in mind, the “right” sample size should be informed by the anticipated conditions under which the larger-scale trial will be conducted.

This does not imply that every possible scenario in the at-scale trial needs to be accounted for in the pilot/feasibility study. Rather, investigators should understand the more salient factors that could lead to differences in both impact and implementation in the at-scale trial and attempt to include these within early-stage studies. To effectively design an informative small pilot/feasibility study assumes an understanding of how an intervention will operate under various circumstances as well as knowing whom one is targeting for the intervention. What are the prototypical characteristics of the intervention, the setting, and the participants and how do these potentially interact to influence the intervention’s impact and implementation? How much variability is anticipated in these characteristics that could influence impact and implementation when scaled? Answering these questions can serve to inform key decisions about the design, delivery, and interpretation of the to-be conducted pilot/feasibility study(ies).

Unfortunately, there are no clear “rules of thumb” or formulaic approaches for an explicit number of individuals or settings required for a preliminary study of a given intervention. Attempts to provide one would be speculative at best, be applicable to a narrow range of conditions, and be inconsistent with the need for a flexible/adaptive approach based upon the complexity of a given intervention designed for a given population to be delivered within a given setting. For instance, an intervention designed for a highly homogenous target population, with minimal technical complexity would require far fewer participants to establish sufficient evidence for scaling compared to an intervention comprised of a larger number of components to be delivered to a wide range of individuals across varying settings. In either scenario, a formal formulaic approach for sample size justification is indefinable. Rather, a sufficient sample size would require evidence to be presented which demonstrates the assumptions regarding the intervention and target population are tenable for the preliminary study, as well as hold for the conditions in the larger-scale trial it [*the preliminary study*] is designed to inform. The “right” sample size for a given preliminary study will, therefore, inherently vary in size from study to study based upon the complexity of the intervention and the characteristics of the target population. In the end, sample size adequacy is best determined within the context of the intervention under study and justified by the broader literature base.

### Recommendation

We recommend intervention designers have clear understanding of how an intervention potentially interacts with individuals and settings and the extent to which such interactions may lead to differences in delivery and outcomes in both the pilot/feasibility study and the anticipated larger-scale trial. There is a wealth of information from published literature on what is reasonable to expect from the delivery of an intervention to a given target population within a given setting. Reasonable and justifiable assumptions are thus readily available and can be incorporated into the design. Small studies employing well-informed sampling strategies can provide valuable information regarding how an intervention is received, delivered, and results in changes in outcomes.

## Challenge — scaling and adaptations

As studies transition to progressively larger sample sizes, an intervention found to be impactful and implementable on a smaller scale in a highly controlled environment may not automatically be similarly impactful and implementable when delivered on a larger scale within a more real-world setting. After undergoing initial testing, an intervention may change in terms of content (e.g., addition/subtraction of behavioral theories, targets, techniques) and dosage (e.g., number and length of sessions). Thus, it is logical to expect an intervention to be altered in some meaningful way based upon pilot/feasibility evidence. How much alterations occur, however, will determine whether additional pilot testing of a revised intervention is required or whether one can move forward with scaling the same/similar intervention in a larger trial.

The question becomes what type of changes require re-testing of the intervention under pilot-like conditions or are so minimal it renders re-piloting unnecessary? The key to an informative pilot/feasibility study is to avoid testing an intervention that may, on the surface, appear similar to the intervention tested in the subsequent larger-scale trial but varies drastically in key areas known to influence impact as well as implementability [8]. It should not come as a surprise that in small studies it is easier to oversee all aspects of the delivery, implementation, and support for an intervention. Likewise, in smaller studies, it is easier to recruit a conducive sample with heightened interest in receiving the intervention. Differences in these factors between the pilot/feasibility study and larger trial can render an intervention found promising based upon feasibility markers and preliminary signal of promise during early testing to show limited or no effects at scale. Below is an overview of common (mis)steps observed in the development and delivery of pilot/feasibility studies that have a high likelihood of influencing the interpretation of markers of both feasibility and preliminary efficacy.

### Common (mis)step — delivering one’s intervention (delivery agent bias)

Many smaller scale, first time-evaluated interventions are delivered by the developer (or their graduate students). The difference in expertise between the developer and a lay practitioner, for a given intervention, can be considerable. Developers are more equipped at dealing successfully with unanticipated situations and scenarios, allowing for beneficial on-the-fly adaptations, rather than reliance upon manualized procedural responses. Delivering one’s intervention, however, is not inherently wrong. In fact, the very first time an intervention is tested, having the developer serve as the deliverer can provide important information about necessary adaptations/modifications. Delivering one’s own intervention becomes problematic when the given pilot/feasibility trial evidence will be used to support a larger, more well-powered trial, not another pilot evaluation of the intervention. Evidence shows when the delivery agent changes from a study author or graduate student during the pilot/feasibility study to a lay practitioner in the larger-scale trial, a sizeable reduction in the intervention’s impact is observed [8]. Delivering one’s own intervention can influence estimates of feasibility as well as potential efficacy of the intervention. It eliminates the ability to evaluate the impact of deviations from intervention delivery protocol (i.e., fidelity) which can prove invaluable during the early design stage when attempting to determine if changes to one or more intervention components is required [10]. This can lead to a lack of understanding of whether the intervention is too complicated to deliver or whether those delivering will do so according to design. Where a highly qualified individual delivers the intervention to a small number of participants during the pilot/feasibility study, one should ask whether they would anticipate similarly skilled delivery in a larger-scale trial and if not, how a change in the delivery agent may influence whether the intervention will retain impact when delivered to a larger number of people.

### Common (mis)step — providing unstainable support for implementation (implementation support bias)

When developers are not the delivery agent, they may provide ongoing, intensive oversight for the implementation of the intervention that is likely impractical in a larger-scale trial. Frequent check-ins with deliverers and in-person attendance at sessions during the early testing of an intervention can prove useful in the identification of unanticipated challenges with the delivery and receipt of the intervention — critical markers of feasibility. This can lead to quickly implementing corrective measures. Such oversight can also improve the overall quality of the delivery and fidelity to the intervention, thereby increasing an intervention’s preliminary signal. As with delivering one’s intervention, this practice is not inherently wrong. This practice, however, can lead to inflated impressions of the signal, as well as a false sense of assurance that those delivering the intervention can do so with a high degree of fidelity. If such support cannot be provided or is simply not realistic in the larger-scale trial, this can lead to a reduced impact of the larger-scale trial [8].

### Common (mis)step — recruiting a conducive sample (target audience bias)

In small studies, it is easier to recruit a conducive, motivated sample. Individuals who are first to respond to advertisements to participate in a study can be fundamentally different to those that require more time to decide about participating — even if they exhibit the prototypical characteristics of the target population. As many behavioral interventions target settings, investigators may approach locations where longstanding relationships already exist, thereby reducing barriers to entry and increasing adherence to the intervention. These issues are analogous to those raised with causal inferences from convenience samples, such that a sample from those sources differ from the general target population of interest on unmeasured confounders and attributes [18, 19]. In the context of preliminary studies, convenience sampling could lead to biased estimates of both feasibility and impact. Likewise, pilot/feasibility studies may test the intervention in a sample that may not reflect the intended target sample, such as testing an intervention in a more well-educated sample [8]. Narrowly selecting participants or settings under which the pilot is conducted helps to maximize the detection of a signal. It also provides misleading information regarding markers of trial and intervention feasibility. Conducive samples may express greater “satisfaction” with the intervention, attend a greater number of sessions, or have fewer barriers to participation than a more representative sample that would participate in the larger trial. These “first in line” effects can lead investigators to believe their intervention has greater impact and acceptability than what is ultimately observed in a scaled trial. These can lead to misguided judgments about an intervention’s readiness to be evaluated in a larger trial. The sample for a preliminary study requires a scientifically defensible rationale and should be based upon the target audience for which the intervention is designed for in the larger-scale trial.

### Common (mis)step — duration of intervention unrelated to impact (intervention duration bias)

Early-stage studies may be designed to evaluate an intervention for shorter periods of time than what is anticipated for the larger-scale trial. For instance, a pilot/feasibility study may last a few weeks, only to be evaluated for substantially longer-durations in the scaled trial. Shorter durations are inversely associated with the impact of the intervention where shorter intervention results in larger effects [8]. Evaluating an intervention under these conditions can lead to inflated estimates of feasibility whereby having to attend fewer sessions over a shorter timeframe may be more appealing and practical to a target audience versus a substantially longer period of time in the larger-scale trial. Greater attendance may result in higher levels of impact of the intervention and change in the signal. This phenomenon may be due to a novelty effect of participants interacting with an intervention, where initial excitement of participating results in greater changes or greater adherence and involvement with the intervention. Conversely, longer exposure to behavior change may result in burnout or a reverting to entrenched habits, leading to reduced impact in the larger-scale trial.

### Common (mis)step — neglecting to repilot

At times, well-designed and delivered pilot/feasibility studies can lead to decisions about completely redesigning an intervention. In these situations, the requisite next step is recursive, and re-piloting the retooled intervention required. The challenge is how to distinguish incremental changes from major reworkings. As mentioned earlier, what constitutes major adaptations or minor tweaks is ultimately left to the investigators and grant review panels to decide. When major changes are necessary, this should not be viewed as “lessons learned” and the changes incorporated into the larger trial for a first-run evaluation. Conversely, it may not be practical, given the constraints of both time and funding, to conduct another preliminary study of a retooled intervention. However, re-piloting rather than wishful thinking is necessary to ensure the investment in a larger-scale trial is based upon strong evidence given the adaptations identified in the pilot.

### Common (mis)step — conflating feasibility with impact

In some instances, investigators may conduct a preliminary pilot/feasibility trial and report on markers of intervention and trial feasibility, solely, and proceed to a scaled more well-powered trial. These can include recruitment numbers, attrition rates, acceptability/satisfaction from the target population about the intervention, and/or qualitative focus groups/interviews. While each of these are critical pieces of information regarding the trial’s performance and scalability, they are insufficient without also collecting information regarding an intervention’s ability to impact primary and/or secondary outcomes. As described in translational science frameworks [13, 14], as an intervention moves progressively across the “maturity continuum”, there is a need to demonstrate that an intervention can be conducted (i.e., implemented) and shows promise for impacting one or more primary and/or secondary outcomes. Thus, just because an intervention can be delivered and is well received does not mean it makes an impact on the hypothesized outcomes [20, 21]. Moreover, if markers of feasibility are collected with one or more of the above common (mis)steps, such evidence would lead to biased interpretations of how an intervention will perform at-scale.

### Recommendations

The aforementioned common (mis)steps are appropriate for *very* early-stage, *first-run* interventions to “develop and refine” procedures. However, these (mis)steps are often done with the results interpreted as the promise of an intervention, only to conduct the larger-scale trial with the features removed and limited impact observed [8]. When incorporated appropriately in first-run interventions, they can serve as useful information for intervention developers in designing a subsequent pilot/feasibility trial. Scaling decisions, however, should not be based upon studies with these features.

The following recommendations are provided:

- Does delivering the intervention yourself or by your graduate students have any impact on the impact of the intervention — if the answer is yes, needs another pilot
- Can the type of support be realistically, logistically, and financially provided in the intervention at-scale — if the answer is no, needs another pilot
- Does the sample included reflect the target population and target setting(s) of the larger trial — if the answer is no, needs another pilot
- Is the length of the intervention in the pilot/feasibility study similar to the anticipated length of the intervention in the scaled trail — if the answer is no, needs another pilot
- Do the results of the pilot/feasibility study suggest major redesigns to the intervention — if the answer is yes, needs another pilot
- Were markers of trial feasibility, intervention feasibility, and preliminary effectiveness collected — if the answer is no, needs another pilot

## Challenge — funding small studies

Well-powered trials require promising pilot/feasibility data but it is unclear if promising pilot/feasibility data be generated in the absence of funding [14]. A major challenge faced by all intervention developers is securing funds for the execution of one or more high-quality early-stage pilot/feasibility studies. From an interventionist perspective, conducting one or more pilot/feasibility studies requires resources, which are limited and competitive [22]. Resource constraints can have unintended consequences and inevitably force decisions to be made about the characteristics of a pilot/feasibility study that can complicate the interpretation of the evidence gathered. Limited resources can result in having to rely upon oneself or students to deliver the intervention or the intervention being evaluated for a much shorter timeframe than anticipated in the scaled trial. Such decisions can inadvertently lead to inflated early-discoveries and false impressions of both feasibility and efficacy, resulting in premature scale-up of an intervention that’s more likely to fail in a larger trial.

There is collective agreement that quality preliminary studies are necessary to make decisions to invest in large-scale trials. This is evidenced in funding announcements from major governmental funders such as National Institutes of Health, the Medical Research Council and National Institute of Health Research in the UK, the National Health and Medical Research Council of Australia, and the Canadian Institutes of Health Research and embedded within intervention development frameworks they [*governmental funders*] endorse [13, 14]. Where funding for such studies can be acquired, however, is difficult. Sources of funding, such as internal institutional/university or foundational grants may prove helpful in supporting early-stage work but are often limited in the total budget and thus unable to fully alleviate resource constraints encountered with behavioral intervention studies. Governmental agencies who predominately fund larger-scale studies are an additional source for support for preliminary studies. In the USA, the National Institutes of Health, the nation’s premier medical research agency, has a number of grant mechanisms to support preliminary studies. Some mechanisms are explicitly designed to produce pilot/feasibility data (e.g., R34) while others are commonly used for such purposes (e.g., R03, R21, Ks).

According to the National Institutes of Health Research Portfolio Online Reporting Tool (accessed January 6, 2021), from 2000 to 2020, the National Institutes of Health invested in an average of 9,226 pilot/feasibility mechanisms (i.e., R03, R21, R34, and K awards) representing an average annual investment of $1.513 billion. Trends in funded applications show that investments in pilot/feasibility studies grew steadily from 2000 to 2008–2009 and since have plateaued for K and R21 awards at ~ 4000 awards/year per mechanism, while declining for small-scale R03s. This is in contrast to the funding of larger-scale trials, most commonly the R01 mechanism, where an average of 28,592 applications were funded each year over the same 20-year time period at an average annual budget of $10.593 billion. More recently, the National Institutes of Health reduced the number of opportunities to apply for early-stage funding by retiring mechanisms that support this work. For instance, the National Institutes of Diabetes and Kidney Diseases recently retired 7 grant mechanisms explicitly earmarked to support pilot/feasibility studies and replaced these with a single mechanism that would only fund up to 3 projects annually [23, 24]. Major funding agencies likely have the most to gain from investigators conducting high-quality preliminary studies and therefore, should also serve as a major source of funding for early-stage studies. However, while opportunities for funding early-stage studies exist, they are far fewer than the large-scale trials that rely upon them to be successful and have been reduced in recent years.

### Recommendation

If we agree that high-quality pilot/feasibility studies are necessary to make informed judgments about investing in a larger-scale trial, additional investments should be made to support them. Moreover, funded early-stage studies should be held to standards that explicitly indicate the elimination of the common (mis)steps described herein. A previous review found that of the “well-funded” pilot/feasibility studies, as identified by stated funding source, that half contained one or more of the common (mis)steps [8]. Although establishing funding opportunities for pilot/feasibility studies is outside the purview of individual research scientists, collectively this group has the ability to advocate for changes to funding structures to provide additional resources for supporting this important stage in intervention design and testing [22]. Thus, additional funding opportunities coupled with standards to assist intervention developers to avoid introducing artifacts that can inflate estimates of initial feasibility and impact are needed.

## Discussion/conclusion

**Fig. 1:**
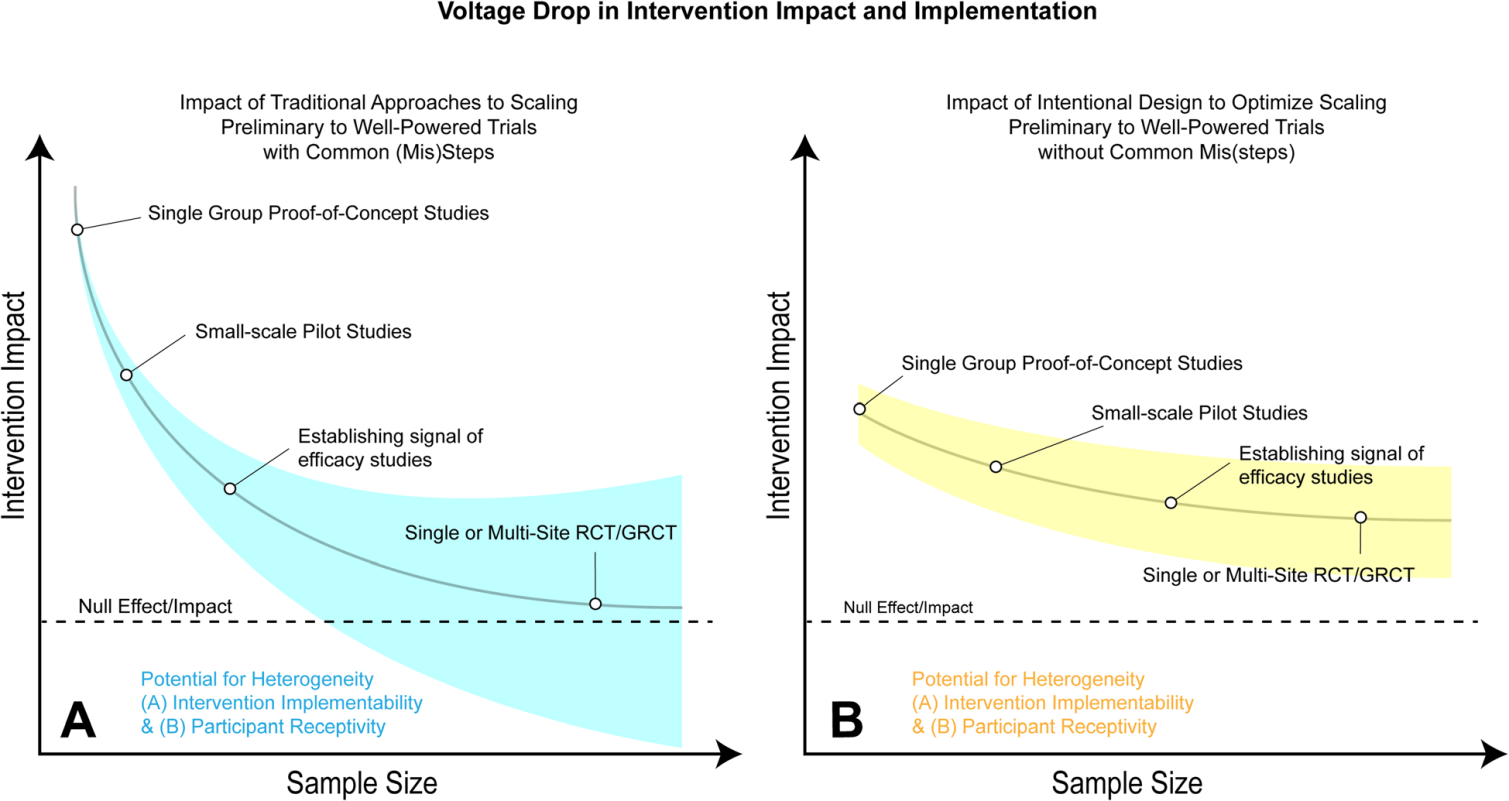
Theoretical voltage-drop in intervention impact from scaling with and without common (mis)steps

Funding intentionally designed pilot/feasibility studies with these considerations in mind will result in the creation of early-stage studies that more readily mimic the conditions and effects to-be observed of the larger-scale trial and optimize the identification of interventions that should and should not be scaled. Intentional design and the removal of these features should also reduce the widely observed voltage drop [25] of the intervention from the initial evaluation through the large-scale trial (see Fig. 1). Panel A of Fig. 1 depicts the hypothetical voltage drop of traditional approaches to intervention development and testing that incorporate one or more of the common (mis)steps mentioned above. As illustrated, the impact of the intervention is substantially large in the early stage of testing (i.e., single group, small-scale pilot), with the slope of the impact curve reduced steeply between each subsequent study to the point of being rendered null in the single/multi-site trial. Moreover, the variability in the delivery and receipt of the intervention is minimized at the early stages given the constraints the common (mis)steps provide early on, with these relaxed and with this the introduction of greater degrees of heterogeneity of implementation and responsiveness at each larger evaluation, all of which contributes to the steep decline in impact (i.e., voltage drop).

Conversely, panel B of Fig. 1 depicts the intentional design of an early-stage pilot/feasibility study without the common (mis)steps. Theoretically, this would lead to smaller impact early on (compared to early-stage studies in panel A), but the slope of the voltage drop would be more gradual and less overall given the smaller-scale studies were designed to mimic the conditions of the larger-scale trial. The variability in implementation and responsiveness would be greater during the early-stage studies, compared to more traditional approaches (panel A), but would experience less growth in heterogeneity across subsequent studies, thereby leading to an impact at the latter stages.

It is important to note that removing common (mis)steps via the intentional design of early-stage pilot/feasibilities studies will not eliminate “null” or “negative” findings in larger-scale trials. Addressing the common (mis)steps should, however, help to reduce null/negative findings that could be avoided and, in hindsight, were apparent. Engineering factors with known influence on both markers of feasibility and preliminary efficacy out of pilot/feasibility studies should reduce unnecessary noise and allow behavioral scientists to focus on more salient underlying causes for either a lack of implementability and/or impact of an intervention at-scale [4]. Ultimately, this would result in a greater number of findings with of substantial scientific merit [26].

In conclusion, pilot/feasibility studies play an integral role in the developmental pipeline of almost every larger-scale trial. Designing an early-stage study that provides evidence of the scientific meritoriousness of conducting a larger-scale trial is, therefore, the primary intent of pilot/feasibility testing from an interventionist viewpoint and a necessity for funding agencies. The stated recommendations are not meant to slow down progress or create cumbersome barriers to scaling interventions. Rather, the recommendations shed light on specific challenges interventionists face in the design and execution of smaller-scale early-stage studies and how these can influence the likelihood of success in a scaled intervention. It is intended that the recommendations provide guidance to speed up incremental science, where higher quality first-run studies are conducted, while simultaneously slowing down premature scale-up, where any evidence is sufficient for scaling.

## Data Availability

NA
